# “The stay here is, of course, not appropriate for an old person”: the perspective of healthcare providers on older patients in the emergency department

**DOI:** 10.1186/s12877-024-05429-9

**Published:** 2024-10-29

**Authors:** Johannes Deutschbein, Andreas Wagenknecht, Gabriela Gilles, Martin Möckel, Liane Schenk

**Affiliations:** 1 Institute of Medical Sociology and Rehabilitation Science, Charité—Universitätsmedizin Berlin, Corporate Member of Freie Universität Berlin and Humboldt-Universität zu Berlin, Berlin, Germany; 2 Division of Emergency Medicine Campus Mitte and Virchow, Charité—Universitätsmedizin Berlin, Corporate Member of Freie Universität Berlin and Humboldt-Universität zu Berlin, Berlin, Germany

**Keywords:** Emergency department, Older patients, Frailty, Qualitative study, Health services research

## Abstract

**Background:**

In aging societies, emergency departments (ED) face an increasing number of older, geriatric patients. Research shows that older emergency patients have a greater burden of comorbidities and a higher risk of adverse events. It has been questioned whether contemporary ED structures can meet the specific needs and characteristics of older patients. Little is known about how professional health care providers perceive and experience ED care for older patients. This study aimed to get insight into the perspective of healthcare providers working with older ED patients and to explore the challenges they experience in their daily work.

**Methods:**

The study used a qualitative research design with a social-constructivist perspective and a Grounded Theory based methodology. Data were collected through qualitative interviews with *N* = 25 healthcare providers from different urban EDs in Berlin, Germany, and adjacent healthcare institutions. Following the Ground Theory approach, categories and central themes were identified, analyzed, and interpreted to gain a comprehensive understanding of the healthcare provider perspective.

**Results:**

The interviews revealed a significant and increasing relevance of geriatric ED patients for healthcare providers. However, there was no shared definition of ‘the geriatric patient’. Most interviewees found ED structures to be inadequate for older patients. They described specific challenges, such as information gathering and safety risks in the ED, as well as an increased use of resources (both time and personnel) when caring for older patients. In addition, specific problems in the collaboration with other professions and institutions were addressed, namely nursing homes, hospital wards, consultations, and the hospital social service.

**Conclusion:**

Healthcare providers experience a structural mismatch between contemporary EDs and the specific needs of geriatric patients. They are aware of the vulnerabilities of geriatric patients and try to compensate for inherent structural shortcomings. Such structures and limited resources often cause practical, organizational, and ethical problems. There is a great need to develop, implement, and evaluate systematic approaches and care concepts that address the specifics of ED care for geriatric patients.

## Introduction

In the light of aging populations, the impact and consequences of increasing numbers of older and geriatric patients for healthcare institutions are widely discussed, including those for emergency departments (ED). EDs represent a safety net as well as a frequently visited site within the health care system which is of particular importance for vulnerable populations such as older people. This is reflected in a disproportionate use of EDs by patients over 65 years of age [1], including a higher proportion of frequent users [2]. The increasing number of older patients has been identified as one driver of ED overcrowding that is a burden on EDs worldwide [3]. Next to the increasing number of older adults, this is also due to the disproportionate number of frequent visits among elderly [2]. However, the vast majority of ED visits by older adults seem to be appropriate, only about 10% can be regarded as ambulatory care sensitive, i.e., could have been avoided by better primary care [4].

Previous research found that ED visits by older adults have significantly worse courses and outcomes than those of younger patients: older ED patients experience longer stays [5], are more often admitted to hospital [1], need to return more often, and have a high risk of adverse outcomes like a functional decline or death [6].

Consequently, contemporary EDs have been criticized in recent years as they are not designed to meet the specific needs of older adults. Evidence on potentially useful care concepts is growing [7], and new guidelines are increasingly published. However, the majority of EDs caring for older patients, especially in Germany, keep practicing the traditional ED model mostly aligned to younger adults with injuries and acute illnesses.

Beyond noting the increasing frequency of ED visits by older patients and beyond debating the appropriateness of ED visits, little is known about the perceptions and experiences of ED practitioners and adjacent healthcare providers who routinely care for older ED patients. Reconstructing the importance of older patients to ED staff and the specific challenges this population causes for personnel’s day-to-day work has rarely been conducted. Only a few studies have inquired the provider perspective, mostly of ED nurses [8–10], none of them came from Germany.

Therefore, this study aimed to qualitatively explore the situation of older ED patients from the perspective of healthcare providers and to analyze the challenges in routine ED care.

## Methods

### Background

The study presented here is part of the mixed-method project EMAAge from the EMANet research network. The consortium is a collaboration of institutions at the Charité – Universitätsmedizin Berlin with expertise in health care research and the provision of emergency and acute care Berlin. All eight Emergency Departments (EDs) of the central district Berlin-Mitte are part of the network, including two EDs of university hospitals and six EDs of general hospitals of different sizes, structures, and ownerships. The largest ED treats almost 60,000 patients per year. The overall aim of EMANet is to set up a sustainable structural and scientific collaboration in order to improve trans-sectoral, multi-disciplinary care of multimorbid patients admitted to (EDs) in Berlin-Mitte. Scientific goals have been the assessment of trans-sectoral health care supply pathways, supply gaps and inadequate resource allocations for multimorbid patients admitted to EDs as well as the identification, development and implementation of improved and cost-efficient health care provision to multimorbid patients with acute conditions [11].

EMAAge is a mixed-methods study focusing on the evaluation of emergency and post-discharge care pathways and patient-centered outcomes of frail, older patients, also called ‘geriatric patients’. As quantitative part, a multicenter cohort study on patients with hip fractures was conducted. Hip fractures are a typical and often dramatic ED indication of geriatric patients [12]. This cohort study was complemented by a qualitative interview study. Here, the perspective of professional health care providers was systematically inquired. In the qualitative interviews, the focus was broadened on the relevance and perception of ‘geriatric patients’ in the ED irrespective of the indication.

The study protocol was registered in the German Clinical Trials Register (DRKS00014273). The ethics committee of Charité – Universitätsmedizin Berlin (EA1/362/16) approved of this study.

### Methodological and theoretical background

Methodologically, the study is based on the logic of Grounded Theory: the analysis is exploratory, aiming at the discovery of “the new” and hence, not strictly oriented on an existing theory that should be verified. However, there are basic theoretical assumptions that guided both the conception of the study and the analysis of data. These assumptions stem from social-constructivism [13, 14] and specifically sociological practice theory [15]. According to this approach, social reality is constructed and reproduced through the practices of social agents. At the same time, agents are bound to (social) structures and internalize them. Practices and social structures form a complex, interwoven, and relatively stable relationship. One way to study this relationship is by analyzing the knowledge and experiences of agents in a specific field. Such data not only provide access to agents’ attitudes and opinions but also to the logic of structures and the logic of agents practicing within these structures. Thus, the study addressed interviewees not only as experts in their profession but mostly experts of their everyday work life in order to access and reconstruct both ED practice and structures.

### Data collection

The data base for this study consists of 25 qualitative expert interviews with professional health care workers (physician, nurses, etc., see Table 1). Sampling focused primarily on ED personnel, i.e., physicians and nurses, that was recruited in six different EDs, including two large EDs of university hospitals. It was aimed to yield a diverse sample regarding gender, professions and disciplines, experience and status, and ED types. Next to ED staff, adjacent health care providers of geriatric ED patients were included: EMS workers, nursing home managers, mobile nursing service managers, general physicians, and geriatricians. Sampling was guided by the idea of “theoretical saturation” [16].

**Table 1:** Characteristics of study participants

	Total, No.
Sex	
Female	10
Male	15
Institution	
Emergency Department	18
Nursing Home	3
General Practice	2
Clinic for Geriatrics	1
Nursing Service	1
Emergency Medical Services	2
Profession	
Senior Physician	4
Head Physician	1
Assistant Physician	7
ED Nursing	8
Head of Nursing	5
Emergency Medical Services	1
Head of Emergency Medical Services	1

*N* = 25; including two participants with dual roles

Interviews were headed with the term ‘geriatric patients’ as the most common expression for frail, older patients in Germany. Referring to the interviewees’ common sense, the term was not defined beforehand. Older patients and elderly were used synonymously.

A semi-structured interview guideline was used that was based on a systematic analysis of literature. It was discussed with colleagues from research and clinicians and adapted throughout the data collection process. The interview guideline was used as flexible as possible in order to create a quasi-natural conversation. Interview questions and stimuli aimed at eliciting experience-saturated narratives and descriptions of personal experiences. The primary aim of the interviews was to give all interviewees room to unfold their own priorities. Throughout data collection, interview strategy and interview guidelines were reviewed and adjusted as required based on previous experiences and gathered information.

All interviews were conducted face-to-face, audio-taped, and transcribed verbatim. They lasted from 40 to 80 min.

### Data analysis

The audio recorded qualitative interviews with health care professionals have been transcribed, pseudonymized, and a thematic overview has been generated. After completing this preparatory process, the interviews have been subjected to qualitative analysis and interpretation. Grounded Theory (GT) served as the backbone of the interpretation [16, 17]. GT is a discovery-led method and ensures openness in the interpretation process. The claim of a representative and comprehensive explanation of a phenomenon is not sought in GT. Rather, the goal is to achieve a conceptual depth of understanding of a phenomenon.

GT offers techniques for obtaining an overview of larger data sets on the one hand, and for conducting detailed, in-depth analyses of individual (interview) sequences on the other. The goal is to gradually generate categories that are not predetermined, emerge from the empirical data and prove to be relevant for understanding the research subject.

According to GT, the material was coded inductively in order to identify recurring themes in the interviews. In the first step, coding was done as openly as possible. In the second step, the codes were grouped and central themes emerged. Increasingly, a thematic focus took place and coding was “more directed, selective, and conceptual” [17]. In the third step, selective coding was employed, i.e., seeking material to place the themes identified thus far on a broader empirical basis. Overall, the analysis followed an iterative process, allowing new insights to influence prior codings, which in turn informed subsequent analyses.

Results were discussed within the research team and in an interdisciplinary qualitative working group to assure intersubjective validity.

## Results

**Fig. 1:**
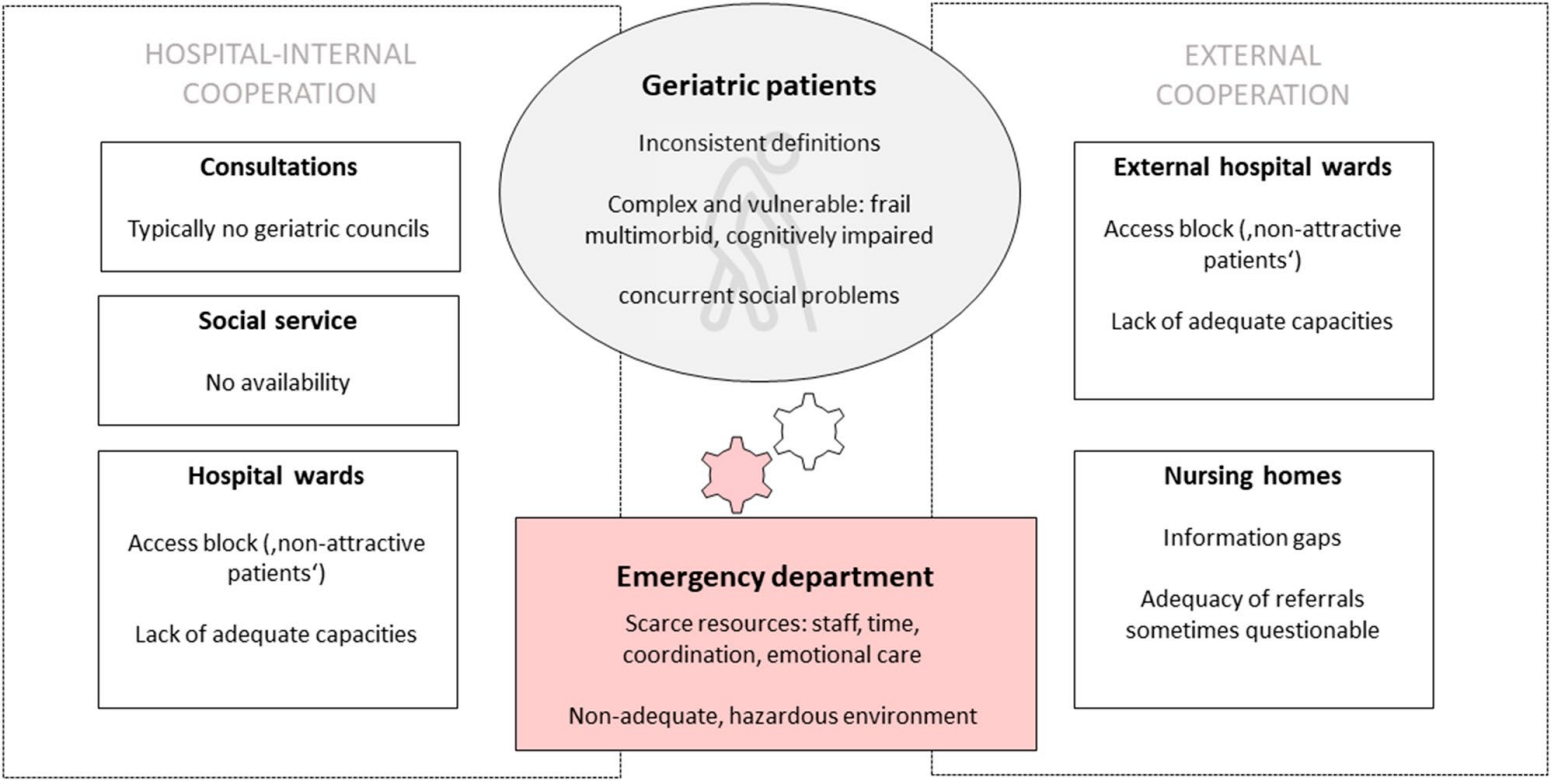
The structural mismatch between the ‚traditional‘ emergency department and geriatric patients

The systematic analysis of the qualitative interviews revealed seven main themes that characterize ED care for geriatric patients from the healthcare provider perspective. These themes could be classified into two main categories. The first one directly addresses the ED patients, their characteristics, and their impact on professionals’ work: the definition of geriatric patients, the quantitative and qualitative evaluation of geriatric patients in EDs, and peculiarities and challenges connected with geriatric patients. The second category of themes addresses the collaboration with other professions and institutions when EDs care for geriatric patients: nursing homes, hospital wards, consultations, and the social service (see Fig. 1).

## Definition of ‘the geriatric patient’

When interviewees were initially asked about their experiences in caring for ‘geriatric patients’, respondents typically raised the question how ‘the geriatric patient’ is defined. No respondent referred to an ‘offical’ definition that was used in ED work routine. Most of the interviewees stated to have no clear definition or they rather use ‘personal’ definitions. Both physicians and nurses mentioned age several times as an obvious but insufficient factor for defining geriatric patients:

*Age is so relative*,* it’s insane. Sometimes we have 90-year-olds who are in top shape and then 60-year-olds who are […] very ill […]. So it’s difficult to set an exact age. (I18*,* ED nurse)*.

And:

*There is certainly an age definition*,* which is mostly 60 plus*,* […] but this is a definition from guidelines and textbooks. At the end of the day*,* I have to say*,* that some 90-year-olds [are] cognitively fitter*,* physically more active […]. Then again*,* there are some at 50*,* 60 that are cognitively on the level of a 90-year-old person with dementia. (I16*,* senior physician*,* ED).*

Instead of age per se, often a combination of age and certain health characteristics was seen as decisive for the definition as a geriatric patient. One physician stated:

*So*,* a lot of the patients we have here […] are returnees. They have received geriatric care or they have so many previous illnesses […] For me*,* that is a geriatric patient. That is*,* old*,* a lot of pre-existing conditions […]*,* and mostly cognitive deficits. (I8*,* assistant physician*,* ED)*.

Another assistant physician provided a comprehensive list of criteria which included the age range of 70–75 years, as well as other factors such as the patient’s overall physical condition, nursing home residency, reduced disease recovery abilities, dependence in activities of daily living, presence of multiple chronic conditions, cognitive status, and rehabilitation potential.

Both physicians included criteria from different areas: organizational (‘returners’), medical and psychological aspects, as well as living and care arrangements, and criteria such as the patient’s potential to participate in a geriatric rehabilitation program.

Another position identified geriatric patients by typical reasons for the ED visit:

*Geriatric emergency patients in our hospital are usually those patients who have fallen. With head lacerations*,* fractures*,* or the like. (I24*,* head of nursing*,* ED)*.

Overall, respondents typically associated the term ‘geriatric patients’ with multimorbidity, cognitive impairments and long-term care dependency, especially living in nursing homes.

## Quantitative and qualitative evaluation of geriatric patients in EDs

ED physicians and nurses mostly shared the impression that geriatric patients are one of the largest patient populations in the ED and that their proportion is increasing, as one senior physician pointedly states: *“we of course have very many geriatric patients*,* and increasingly so” (I10*,* Senior physician*,* ED).* Not just socio-demographical reasons, but also medical reasons for the high and increased incidence were outlined. For example, that *“you just get sick more often in old age” (I22*,* assistant physician*,* ED)*.

At the same time, ED personnel mostly considered EDs to be inadequate for the specific conditions and needs of older patients. The ED was seen as a place potentially causing a mental burden and a source of subsequent health risks. Risk factors regularly mentioned by the interviewees were extended length of stay and waiting times, heightened stress level caused by noise, restlessness and many unfamiliar people. This could lead to a general psychologically exhausting situation, the feeling of helplessness, discomfort, partial resentment, frustration, but also adverse events such as falls or delirium. Throughout the interviews, the non-match between ED environment and elder patients was addressed by nurses and physicians:

*The stay here in the emergency department is of course not appropriate for an old person*,* right? So*,* this is not a pleasant environment. (I9*,* ED*,* nurse).*

And:

*An ED is not a good place for geriatric patients*,* yet one where they are very*,* very common. (I19*,* assistant physician*,* ED)*.

The ED environment was regarded as a hazardous environment, not only but in particular for patients with dementia:

*Especially the older ones*,* who don’t know what’s going on now*,* wonder why am I lying here on the stretcher all the time and then they climb over the bed rail and then you have the next fall. (I19*,* assistant physician*,* ED)*.

A senior physician made a similar, yet more dramatic statement regarding patients with dementia:

*Of course*,* being transported by an ambulance through half the city is also an enormous burden for the patients*,* and being taken out of their familiar environment*,* especially for patients with dementia*,* is really throwing them back. It is*,* I believe*,* really a traumatic experience for them. (I10*,* senior physician*,* ED)*

A physician formerly working in an ED and now in a geriatric rehabilitation clinic further mentioned that some of his patients experienced an ED stay as a “shock”. Patients returned from the ED sometimes in a worse shape than before, having developed a delirium.

*Due to the structures of the emergency room itself*,* that is the problem […] However*,* I also experience that people suddenly become delirious here. So*,* they are call from the emergency department and say no*,* the patient is totally fine and accessible*,* but then they come to us and suddenly go completely crazy. (I20*,* senior physician*,* clinic for geriatrics)*

## Working with older patients in the ED – peculiarities and challenges

According to ED physicians and nurses, the population of elderly patients poses special challenges for the everyday work practices of the ED personnel.

First, information gathering for anamnesis, diagnosis, and treatment decision was complex and difficult to obtain. ED personnel described the situation as follows:

*They can’t tell you anything about their health. (I19*,* assistant physician*,* ED)*.

And:

*They can not even describe their symptoms properly. (I9*,* ED nurse)*.

And:

*Patients in old age are naturally more likely to be cognitively impaired*,* are often not well-informed about their own medication and pre-existing conditions and*,* therefore the administrative effort of obtaining information is also disproportionately higher than with young patients. (I22*,* assistant physician*,* ED).*

When patients were not able to provide reliable information about their health status, symptoms, current medication, or past illnesses, ED personnel often needed to identify additional sources such as relatives, nursing home staff, legal guardians, or their general physicians. The problem was increased by a lack of systematic, centralized patient files.

*People are in so many hands and nobody seems to have a real overview of them/ that no one has a real overview of them*,* that’s how I feel. And then they come to the hospital and for the first hour*,* you are just busy somehow trying to gather information about this patient. (I19*,* assistant physician*,* ED)*.

Second, both physicians and nurses emphasized a greater need of older patients for nursing care, including emotional care.

*In all aspects*,* they mostly need support*,* right? Whether it’s drinking*,* repositioning or going to the toilet. Or simply mental support*,* I think […] they need more than younger people. It takes more care. (I17*,* ED nurse).*

Nurses described the specific emotional needs of older patients, including the need for physical contact and communicative needs. Emotional needs were perceived in older patients in general, but especially in patients with dementia.

*Sometimes*,* only to hold the hand […] three minutes are well invested*,* […] if one would also look at it economically*,* right? Because*,* if I have to keep looking ten times to see if they’ve gotten up or not*,* it’s better to just try and radiate calmness*,* right? […] So*,* they become calmer and calmer and then they don’t escape us so quickly*,* right? (I7*,* ED nurse)*.

ED nurses expressed their responsibility to care for emotional needs, and interpreted their strategies as a way to avoid further risks and handle scarce resources in the ED. However, many nurses described their struggle to fully live up to their own expectations given the current ED structures:

*And even the time is missing somewhere to say you take some time for the patient*,* or accompany the patient to the toilet*,* talk to the patient briefly. Nevertheless*,* you are a nursing professional*,* that means consultation and all of this gets lost somewhere. You always try to do damage control and that is at some point*,* I think*,* something that is frustrating*,* because you don’t have the time to do with the patient what you actually learned back then. (I13*,* ED nurse)*.

The third reason why the treatment of older patients often was more difficult and time consuming was an increased medical complexity of geriatric cases.

*In old age*,* they are also often multimorbid*,* so […] they can also be difficult. Even if they only come with similar clinical pictures [as younger patients]*,* there are of course also resulting complications due to the comorbidities of the patients. (I15*,* senior physician*,* ED)*.

Clinical pictures of geriatric patients were also described as ambiguous and prone to misinterpretations:

*When the patient is asked*,* ‘Do you have chest pain?’ he says*,* ‘Yes.’ However*,* he also complains of stomach pain*,* pain in the big toe*,* as well as pain in the ears and hair. So*,* everything is completely vague and difficult to grasp. Then*,* you might do some laboratory tests*,* which reveal significantly abnormal results. Yet*,* it is unclear whether these are acute or a chronic decompensation. (I10*,* senior physician*,* ED)*.

For some interviewees, geriatric patients challenged the traditional ED perspective that clearly differentiates acute problems from chronic conditions and feels responsible only for the acute problem:

*I think*,* this concept [acute problems] is changing. Maybe a maximum of 10% are acute and the majority of geriatric emergency patients are […] internal @polytrauma@*^1^,* […] chronic*,* long illnesses that you cannot treat so fast. (I13*,* ED nurse)*.

In addition, interviewees discussed the frequent problem of older patients with so called nonspecific complaints such as deteriorations of the general condition:

*They often come to the emergency department with @nonspecific complaints@*,* and supposed results can also sometimes be false friends. Overall*,* it is more lengthy*,* more complicated and more demanding to make a concrete diagnosis. (I22*,* assistant physician*,* ED).*

Medical complexity resulted in a comparatively high number of diagnostic procedures:

*You always have to make a relatively broad spectrum of diagnostics and of course have several things in the back of your mind (I9*,* ED nurse)*.

Complexity was additionally caused by considerations on end-of-life care that some physicians expressed. They described ethical doubts when it came to complex diagnostics or treatments for seriously ill patients:

*When we get a patient in a really bad condition*,* which is not uncommon*,* we stand there in the shock room with maximum equipment*,* the anesthesia and intensive care team*,* and then we ask ourselves how far do we want to*,* or should*,* or have to go with a 90-year-old*,* right? That’s a huge problem. (I16*,* senior physician*,* ED)*.

Here, physicians argued that they were missing (proper) patient directives to guide them:

*Of course*,* if you have no kind of therapy limitation written down and you can’t reach a legal guardian […] then you tend to do everything*,* if you don’t have a negatively documented patient’s will. Of course*,* that’s difficult*,* yes*,* ethically it’s questionable. (I10*,* senior physician*,* ED)*.

## Collaboration with additional health care providers

In the course of the entire treatment of the older or geriatric patient, interdisciplinary collaboration between different health care providers takes place before, during and after the ED stay. This collaboration and a multitude of communication and organizational problems emerged as another main topic of the interviews. Four relevant aspects of inter-organizational and inter-professional-cooperation were discussed by the interviewees.

### Nursing homes

In nearly all interviews the cooperation with nursing homes became a subject, mostly framed as a problematic one. Geriatric patients were often associated with nursing home residents and interviewees emphasized the relevance ED patients referred by nursing homes. Collaboration was described as difficult, pointedly called a *“nursing home – ED – ping-pong-game” (I1*,* assistant physician*,* ED)*.

First, some interviewees questioned the adequacy of ED referrals from nursing homes. They reported to have patients from nursing homes with only trivial reasons to visit such as the exchange of catheters, minor injuries, or general nursing care neglect.

*And then you constantly have these elderly people who have fallen*,* but obviously*,* nothing is wrong with them. You always wonder why they are in the emergency department now. They are only in the emergency department because the caregivers on-site refused to take responsibility and make that decision themselves. (I23*,* head physician*,* ED)*.

Next to insecurity and the problem of accountability, respondents assumed shortage of staff and insufficient qualifications as reasons for inadequate transfers from nursing homes. However, respondents expressed understanding for the precarious situation in many facilities and sought differentiation between problematic and well-functioning nursing homes:

*Of course*,* I also experience the varying quality of care facilities […] We shouldn’t deceive ourselves*,* it’s very different and it always depends on who has time and also somehow the sense of duty to take responsibility and to take care of things. (I23*,* head physician*,* ED)*.

Second, the communication with nursing homes was often experienced to be difficult, time consuming, and unsatisfying. Critical background information was often missing:

*The patient arrives*,* and they’re simply not doing well*,* yes*,* and then there’s three sentences or three words from the nursing home*,* you can’t do anything with that*,* right. You want to know*,* have they been feeling bad for weeks*,* for years*,* do they have shortness of breath*,* have they vomited*,* what have they eaten*,* why haven’t they eaten. This information is almost never passed on. (I8*,* assistant physician*,* ED)*.

When transfer forms were used, their quality was described as *“very different*,* they are usually also filled out*,* but not always completely up to date.” (I24*,* head of nursing*,* ED)*.

Telephone calls with nursing homes to gather necessary information were described as typical but sometimes arduous since competent contact persons were hard to find:

*I have really experienced that you call and are put through four times until you are with someone who speaks German at all and who then just says the whole time: I am new here*,* I don’t know anyone here. (I19*,* assistant physician*,* ED)*.

And:

*Sometimes*,* when you want to make a phone call*,* you meet leasing staff who don’t know the patient that well. (I15*,* senior physician*,* ED)*.

Nursing home staff, in turn, reported less but also some problems in the collaboration with EDs. The role of EDs in healthcare for geriatric patients was largely appreciated. Decision-making on transfers to the ED was described as cautious and systematic:

*We have emergency plans*,* we have extensive care already in advance with case discussions and the like*,* so that in certain situations we no longer have to call an ambulance in. (I3*,* head of nursing home)*.

Nursing home personnel also expressed their sense of responsibility towards their residents and their awareness of the risks of ED transfers:

*We try to reduce hospitalization and emergency department visits as much as possible*,* because we also know that our residents can be best cared for […] in their familiar environment. And it is also always a stressful situation for us to send someone to an emergency department. (I3*,* head of nursing home)*.

Communication with EDs was described as sometimes problematic in a different way from the nursing home perspective:

*And this sheet disappears principally. […] It usually gets lost in the rescue centers*,* is no longer found there and if the patient then has to go to a ward*,* they have no information at all. (I5*,* head of nursing home)*.

Transfers from the ED back to the nursing home could be problematic, too:

*The only problem is when they don’t call us. When Mrs. Muller suddenly shows up again at eleven o’clock at night. Then*,* it’s not a big problem anyway*,* […] we just don’t know what’s going on. You call the emergency department and no one can tell you. (I2*,* head of nursing home).*

### Hospital wards

Other key challenges were associated with discharging patients from the ED. Admission to a hospital ward was often described as only semi-appropriate but the safest option. Inpatient admission, however, was associated with an intensified access block for older patients:

*To put it in a simple way*,* there are attractive patients for the ward and unattractive one. And for the unattractive ones*,* you have to make more of an effort to get a bed there. (I15*,* senior physician*,* ED)*.

In particular, older patients with certain conditions were seen as ‘unattractive’:

*That’s really the nursing home resident with an antibiotic-resilient germ*,* with advanced dementia. You just can’t get rid of him. […] For such patients you sometimes phone for two hours until you then find someone who has mercy. (I10*,* senior physician*,* ED)*.

When general hospital wards claimed to have no capacity or to be not appropriate for an old patient, one possible solution was referral a geriatric rehabilitation clinic or unit. However, geriatric beds are scarce and they have their own downside:

*The prerequisite is that there is a potential for improvement in order to be able to transfer the patients at all. They don’t usually benefit from two or three nights*,* but from a longer program with mobilization and so on. (I22*,* assistant physician*,* ED)*.

However, not every geriatric patient has a positive prognosis for rehabilitation and many patients are hesitant to visit such a clinic for several weeks. Respondents often expressed their discomfort with the options they had to discharge their patients, especially when sending them back home.

*Where you have a bad feeling*,* if you would discharge them back home without having a bit more care [social support]*,* right? (I7*,* ED nurse)*.

These vulnerable patients, often living alone in their own apartment, were described as helpless and endangered:

*And if they are not admitted as inpatients*,* […] they would need a mobile nursing service. […] How to get a mobile nursing service*,* […] these patients don’t know and they can’t sort it out on their own*,* because they are already in such a desolate state that if you send them home*,* they come back after three days with exactly the same problem. (I13*,* ED nurse)*.

In many cases, a lack of adequate places or institutions to transfer geriatric patients to was perceived.

### Consultations

Collaboration with geriatric or other specialist consults was another topic in the context of cooperative work. When asked, if geriatric consultations were used in the ED, almost all respondents denied:

*I have a complex geriatric clinical scenario and would like an assessment*,* what we should do*,* not because of the disease*,* but because he is so old and has so many pre-existing conditions. This is definitely not happening. […] You ask the respective specialist who is responsible for the disease that is currently in the foreground. […] But a geriatric consultation doesn’t exist. (I8*,* assistant physician*,* ED)*.

If consults occurred, they were sent to medical specialties such as cardiology or neurology. However, some respondents approved of the idea to consult geriatricians:

*So I think it’s definitely a good idea that the patients are not only seen by trauma surgeons*,* because […] they have little in mind than the bones […]. And to look at them [the patients] a bit more holistically […] and also to ask the question why the patient even fell […]. (I19*,* assistant physician*,* ED)*.

However, the holistic perspective geriatricians could provide, was not always seen as part of ED care:

*I think we actually lack the time to really do it adequately in the emergency room. I don’t think […] it is the basic task either. I rather think that the basic task is to recognize what kind of problem the patient has and to decide whether it has to be solved as an outpatient or as an inpatient*,* to make the initial diagnosis and therapy accordingly and then to transfer the patient. (I15*,* senior physician*,* ED)*.

### Social service

Many of the interviewees reported that problems of older or geriatric patients had to be described as social problems, i.e., a lack of social support at home, the inability to care for themselves or self-neglect.

*You tell the patient*,* yes*,* as a matter of fact you would need a mobile nursing service. This is a typical sentence that you hear like 15 times when some physician stands next to a bed. (I13*,* ED nurse)*.

However, ED staff is not qualified nor has it the resources to organize such an outpatient service. This falls within the scope of duties of social services. When asked about the hospitals’ social service, all but one respondent said that there was no collaboration:

*None of the social service ladies are somehow responsible for us as an emergency department. None. So*,* it is purely personal contact. (I23*,* head physician*,* ED)*.

*I’ve honestly never had anything to do with the social service here*,* I don’t know if they cooperate with the emergency department at all. I don’t have a number that I could call. (I19*,* assistant physician*,* ED)*.

If collaboration with the social service happened, it was mostly informal, based on personal contacts and individual action. There was no official, institutionalized, and formalized way of cooperating. In the context of this issue, it was regularly expressed that the availability of social service in the ED setting would be desirable. The lack of systematic recognition of social problems was, for example, perceived as a reason for unnecessary return visits:

*The guiding principle should actually be that every patient only leaves the hospital when their social support is also ensured. Otherwise the probability is very*,* very high that he will visit another emergency department in a very short time. (I1*,* assistant physician*,* ED)*.

## Discussion

In this study, we analyzed how professional healthcare workers, mostly ED staff, perceive and experience ED care for geriatric patients. ED physicians and nurses came from different types of hospitals and EDs, but no ED was part of a specialized program for older patients such as a geriatric trauma unit. Therefore, our data represent the provider perspective on the situation of older, frail ED patients under the conditions of the still prevailing traditional ED model. It can be described as mainly focusing on “single, rapidly developing, immediately reversible problems” [18]. This traditional ED model seems to be challenged by the problems and needs of frail, older patients on various levels.

Our data validate previous research that providers mostly experience a poor fit between ED structures and older patients. The issues identified here as most relevant to providers confirm and extend results of similar, qualitative studies [8–10, 19].

Healthcare professionals found that most ED visits by geriatric patients were appropriate and necessary, unlike many visits by younger patients. This is in line with previous studies [6]. While geriatric patients were regarded as a large patient population with frequent and appropriate ED visits, there was no common and clear understanding of who should be regarded as a geriatric patient. Accordingly, no interviewee reported that frail, older patients were systematically identified as a special population with specific needs or risk factors. Consequently, treatment guidelines or SOPs for geriatric patients were unknown. This might be traced back to the traditional ED model which focuses on specific acute conditions rather than certain populations with general vulnerabilities. Studies investigating the potential of screening tools to identify geriatric patients in the ED have led to mixed results [20]. ED screening tools that have been developed in recent decades to identify geriatric patients and to risk stratify them, mostly failed to prevent adverse outcomes [21]. The feasibility to use additional screening instruments in crowded EDs is questionable. However, making the recognition of geriatric vulnerability dependent on individual views and situational circumstances bears great risks for patients’ safety.

The lack of systematic identification can be seen as one underlying reason for the challenges that geriatric patients pose for healthcare providers and the ED as an institution.

1. Geriatric patients come with more complex problems and needs that require more time and effort both from nurses and physicians. Khilgren et al.‘s study highlighted that effective care in the ED, from a nursing standpoint, hinges on comprehending the needs of older patients, which often deviate from the department’s typical tasks and capabilities. The prioritization of medical procedures and the time-demanding execution of daily routines further complicate the quality of care [10]. We identified similar conflicts between professional ethics and ED operations in our interviews, extending to physicians as well. Interviews from our study highlighted the importance of emotional support for older patients in EDs. These emotional dynamics involve not just patients, but also affect healthcare professionals. The struggle to manage this complexity within time and resource constraints was noted across various studies [8–10]. Our results underscore the significance of emotional labor in nursing care, supplementing the established value of ‘caring conversations’ [22, 23]. This goes beyond managing patients’ negative emotions due to waiting, as seen in previous research [10, 24]. The challenges posed by older patients in EDs, such as information and communication issues, differentiating between acute and chronic problems, and the need for support, are further exacerbated when patients have cognitive impairments or dementia. Therefore, many interventions and care concepts addressing older patients’ needs, specifically focus on those with dementia [25]. As a specific form of complexity, adequate care for old patients with advanced illnesses was discussed. Some physicians described difficult decision making on the extent of treatment, problems with identifying the patient will, and internal conflicts. Similar problems have been identified in two studies from the US [26, 27].
2. Complexity of geriatric cases can be caused or exacerbated by social problems. Older patients visiting the ED often have underlying social problems such as lacking structures of social support in daily living or living in hazardous arrangements. The current structure of the ED lacks both the necessary resources and expertise to address these issues. However, hospital social services have the potential to handle such problems, and some interviewees acknowledged the significance of collaborating with social services. Nevertheless, regular work relations with hospitals’ social services hardly existed, even though research shows the general importance of social services and their capability to take social aspects into account for preserving the successful management of care trajectories - beyond EDs [28] but also for ED patients [29]. Geriatric patients represent only one population where EDs are confronted with primarily or underlying social problems. To meet this phenomenon the concept of social emergency medicine has been suggested, gaining increasing attention [30]. While this idea is often discussed in relation to problems like homelessness or violence, it also seems promising for the social care problems of older ED patients.
3. ED care for geriatric patients often entails a greater need for collaboration with other health care providers, especially in connection with transitions. Collaboration and coordination between EDs and primary care is important in general [31], and even more so for geriatric patients [32]. In our study, hospital wards and nursing homes were discussed as the most relevant partners. ED staff reported on increased problems to find hospital beds for geriatric patients. The general and frequently discussed ED problem of access-block [33, 34] seemed to be even greater when geriatric patients were involved. This was ascribed to their ‘attractiveness’ for wards and the lack of adequate hospital resources. As a result, ED staff, mostly physicians, had to invest enormous effort and time to organize the transition, patients’ ED stays were artificially lengthened. On a cultural and economic level, it was revealed that old or geriatric patients seem to be found at the bottom of the informal hierarchy of patients throughout the healthcare system. One of the most pressing issues for interviewees were patients from nursing homes and the collaboration with nursing home staff. Transfers between nursing homes and EDs are common and have been described as prone to frictions before [35]. Although a relatively small group of older adults and a population that is under medical observation 24/7, nursing home residents account for a remarkable share of visits and are overrepresented in their age group [36]. Previous studies have questioned the appropriateness of emergency admissions from care facilities [37]. To reduce the number of avoidable admissions there are promising approaches such as mobile geriatric teams that visit nursing home residents in urgent care situations [38], and programs to train nursing home staff on emergency management [39]. From the provider perspective in our study, nursing home transfers seemed sometimes avoidable but problems in the cooperation with facilities was of greater relevance. Communication was described as often arduous and information gaps as common. This problem has been shown in both qualitative and quantitative studies before [40–43]. A first step to improve transfers seems to introduce standardized transfer forms [44]. However, many problems in the collaboration with nursing homes were ascribed to fundamental, structural shortcomings within the long-term care system and need to be addressed on a systemic level.
4. From the healthcare provider perspective, the mismatch between traditional EDs and geriatric patients can pose a patient safety issue: the ED is not only unfit to meet those patients’ specific needs, but it can also be a hazardous place for geriatric patients, and again especially patients with dementia. Structural characteristics of EDs such as limited nursing resources or a lack of quiet and safe spaces were described as factors that could contribute to deterioration and complications beyond patients’ original health problem. Although awareness of these risks was pronounced security arrangements were rather situational than systematic. They often seemed to be dependent on advantageous conditions such as good staffing situations or free room capacities. Internationally, different systematic approaches to provide a safe ED environment for geriatric patients have been discussed. One important dimension are fundamental changes in ED designs, including the integration of special zones for older patients [45]. Even the establishment of specific geriatric emergency department units has been suggested [46]. In addition to improvements in the physical environment of EDs, the education and training of geriatric expertise among ED physicians and nurses seems to be of the utmost importance [47].

### Limitations

This study, the first of its kind in the German context, has strengths and weaknesses that need to be taken into account when evaluating its results. As a qualitative study, results are based on individual perceptions and experiences. They represent the subjective perspective of professional agents on the situation of older ED patients. This is a key element of social reality which also provides access to social structures. However, this study lacks the patient perspective which is needed for a holistic picture.

A strength of this study is the diversity of its sample: it comprises interviewees from both key professions, nurses and physicians, and professionals adjacent to EDs. This helped to receive a broad picture of the professional perspective on older ED patients. In addition, interviewees were recruited in six different EDs with a broad spectrum of structural differences (size, provider institution). Diversity was also reached regarding gender, discipline, hierarchical position, or experience. Thus, there seems to be largely empirical saturation regarding ED staff, for adjacent providers the sample size might be too small. There are limitations regarding the scope of the study which was conducted in an urban setting in Germany. Results cannot easily be generalized to small-town or rural areas, nor to other countries and their different healthcare systems.

## Conclusion

EDs and ED healthcare providers are confronted with an ever-increasing importance of older, often frail patients. EDs serve as a safety net for this vulnerable patient population. They have to step in when problems arise and often compensate the deficits in other healthcare sectors. ED staff is aware of older patients’ specific characteristics and needs and tries to meet them. However, within the structure and logic of the traditional ED model, caring for older patients often causes difficulties and problems in ED staffs work routines. There seems to be a structural mismatch between the specifics of geriatric patients and the traditional ED model which causes hazards for patients and practical, organizational and ethical problems for ED staff. Systematic changes of ED structures and collaboration with other healthcare providers seem to be necessary. Acceptance, feasibility, and effectiveness of suggested concepts for geriatric ED care and their implementation need to be further studied.

## Data Availability

Interview data analyzed during this study are included in this published article in form of quotes. The complete interview transcripts generated during the current study are not publicly available due to reasons of confidentiality. Individual reasonable requests for data should be addressed to the corresponding author.

## Footnotes

1 spoke with a laugh

## Abbreviations

ED: Emergency Department
EMS: Emergency Medical Services
GT: Grounded Theory
SOP: Standard Operating Procedure
